# Are Predictors for Overall Mortality in COPD Patients Robust over Time?

**DOI:** 10.3390/jcm12041587

**Published:** 2023-02-16

**Authors:** Noriane A Sievi, Jerome Sepin, Maurice Roeder, Thomas Brack, Martin H Brutsche, Martin Frey, Sarosh Irani, Jörg D. Leuppi, Robert Thurnheer, Christian F. Clarenbach, Malcolm Kohler

**Affiliations:** 1 Department of Pulmonology, University Hospital Zurich, 8091 Zurich, Switzerland; 2 Department of Biostatistics, University of Zurich, 8006 Zurich, Switzerland; 3 Pulmonary Division, Cantonal Hospital of Glarus, 8750 Glarus, Switzerland; 4 Pulmonary Division, Cantonal Hospital of St. Gallen, 9000 St. Gallen, Switzerland; 5 Pulmonary Division, Clinic Barmelweid, 5017 Barmelweid, Switzerland; 6 Pulmonary Division, Cantonal Hospital of Aarau, 5001 Aarau, Switzerland; 7 University Clinic of Internal Medicine, Cantonal Hospital Baselland, University of Basel, 4410 Basel, Switzerland; 8 Pulmonary Division, Cantonal Hospital of Münsterlingen, 8596 Münsterlingen, Switzerland

**Keywords:** COPD, mortality, predictors, longitudinal

## Abstract

(1) Background: Mortality is a major outcome in research on chronic obstructive pulmonary disease (COPD) with various predictors described. However, the dynamic courses of important predictors over time are disregarded. This study evaluates if longitudinal assessment of predictors provides additional information on the mortality risk in COPD when compared with a cross-sectional analysis.; (2) In a longitudinal, prospective, non-interventional cohort study including mild to very severe COPD patients, mortality and its various possible predictors were annually assessed up to seven years.; (3) Results: 297 patients were analysed. Mean (SD) age was 62.5 (7.6) years and 66% males. Mean (SD) FEV1 was 48.8 (21.4)%. A total of 105 events (35.4%) happened with a median (95% CI) survival time of 8.2 (7.2/NA) years. No evidence for a difference between the raw variable and the variable history on the predictive value for all tested variables over each visit was found. There was no evidence for changing effect estimates (coefficients) across the study visits due to the longitudinal assessment; (4) Conclusions: We found no evidence that predictors of mortality in COPD are time dependent. This implies that cross-sectional measured predictors show robust effect estimates over time and multiple assessments seem not to change the predictive value of the measure.

## 1. Introduction

Mortality is one of the major outcomes in research projects on chronic obstructive pulmonary disease (COPD), and the fact that COPD is the fourth leading cause of death worldwide [1] is widely mentioned in presentations and papers on COPD. Mortality caused by COPD seems to be underestimated as a consequence of the missed diagnosis of COPD and underreporting on death certificates due to comorbid conditions such as coronary artery disease that may interfere with the accurate determination of the cause of death [2]. Furthermore, the increasing ageing population and the exposure to known risk factors lead to the assumption that the number of deaths due to COPD may accelerate. In a large study with over 21,000 COPD patients followed up over 11 years, overall life expectancy was 8.3 ± 6.8 years shorter than in the general population with a 3.5-fold higher all-cause mortality [3]. Beside primary prevention such as reducing tobacco exposure and decreasing contact with noxious gases and biomass fuels, there are pharmacological treatments that might reduce COPD mortality [4].

Various predictors related to mortality in patients with COPD were described in the literature, such as dyspnoea, airway obstruction, exercise capacity, or body mass index (BMI) [5]. Furthermore, predictive indices such as the BODE index [6] were suggested to enhance the prediction of mortality in COPD because of its multidimensionality. Despite the various factors known to predict mortality, it remains challenging for physicians to provide an individual prognosis. In most of the studies, predictors were solely assessed at baseline and assumed that the predictive value remains constant over time. The dynamic courses of predictors over time such as lung function or exercise capacity are disregarded in the current concepts. Therefore, we investigated the overall mortality in a cohort of COPD patients and yearly assessed various predictors to evaluate if longitudinal assessment of possible predictors provides additional information on mortality risk as compared with a cross-sectional analysis.

## 2. Materials and Methods

### 2.1. Subjects

In seven pulmonary outpatient clinics in Switzerland, patients with mild to very severe COPD were recruited for the prospective, observational cohort project “The Obstructive Pulmonary Disease Outcomes Cohort Study (TOPDOCS)”. Patients were initially scheduled for three annual study visits (some patients extended their participation up to seven years) where the assessment of predictors took place. Patients were followed-up by phone or medical records at least until death, loss of follow-up, or until October 2020 to assess mortality. The recruitment period lasted from October 2010 to April 2014 with annual measurements until December 2016.

Patients were eligible when aged between 40 and 75 years at inclusion with a confirmed COPD diagnosis according to Global Initiative for Chronic Obstructive Lung Disease (GOLD) guidelines [7]. Patients suffering from mental or physical disability precluding informed consent or compliance with the protocol were excluded. In case of a COPD exacerbation, patients were included in the study or called up for follow-up visits with a delay of at least 6 weeks.

The study was conducted in accordance with the declaration of Helsinki and all subjects gave written informed consent to participate. The Ethics Committee of the Canton of Zurich approved the study (EK-ZH-NR: 1734 and 2011-0106) and the study is registered at www.ClinicalTrials.gov (accessed on 15 February 2023), NCT01527773.

### 2.2. Measurements

#### 2.2.1. Physical Capacity

The 6 min walking distance (6MWD) was annually assessed according to the American Thoracic Society (ATS) guidelines [8]. The 6 min walking test (6MWT) was performed on a 75 m indoor track, and patients were told to walk as far as possible within six minutes. Oxygen supplementation was used if required. 

#### 2.2.2. Respiratory Variables

Standard pulmonary functional testing was performed according to ATS/ERS guidelines [9,10] to measure forced expiratory volume in one second (FEV_1_), forced vital capacity (FVC), and residual volume to total lung capacity (RV/TLC) ratio. Only values after bronchodilation were reported. Disease severity was assessed by spirometric GOLD stages I–IV [11]. 

#### 2.2.3. Blood Gas Analysis

Daytime arterial blood gas analysis was performed to assess partial oxygen pressure (PaO_2_) after 5 min of rest (ABL 700 series blood gas analyzer, Radiometer, Copenhagen). Measurement was performed without supplemental oxygen.

#### 2.2.4. COPD-Specific Health Status

The COPD Assessment Test (CAT) was used to quantify the impact of COPD on a patient’s health [12]. It comprises eight questions rated between 0 and 5, summed up to a total score between 0 and 40 points. Total scores of 20 or above indicate a high impact level with an estimated minimal important difference (MID) of 2 points [13]. The severity of dyspnoea was assessed using the modified Medical Research Council (mMRC) scale [14].

#### 2.2.5. Comorbidities and Survival

Various relevant and most frequently observed comorbidities (i.e., atherosclerosis, arterial hypertension, cor pulmonale, chronic heart failure, diabetes, coronary artery disease, lung cancer, chronic renal failure, or pulmonary hypertension) were assessed at baseline by review of the documented medical history and derived from their treating physicians. To classify comorbidities, the International Classification of Diseases—Tenth Revision [15] was used. 

Survival was assessed by personal phone contact or medical records at the time point of work up for analysis. If patients were lost to follow-up, we either called their next relatives or the general practitioner to receive actual information on survival.

#### 2.2.6. Exacerbation History

An acute exacerbation (AE) was defined as an increase in patient’s dyspnoea, cough and/or sputum with prescription of antibiotics and/or corticosteroids. Severe exacerbation was determined as hospital admission due to AE. Annual acquisition of number of AEs during the preceding year was performed [16]. To get the most accurate information on AE, patient reports were compared with documents from the general practitioner, pulmonologist, and hospital. 

### 2.3. Data Analysis and Statistics

**Figure 1:**
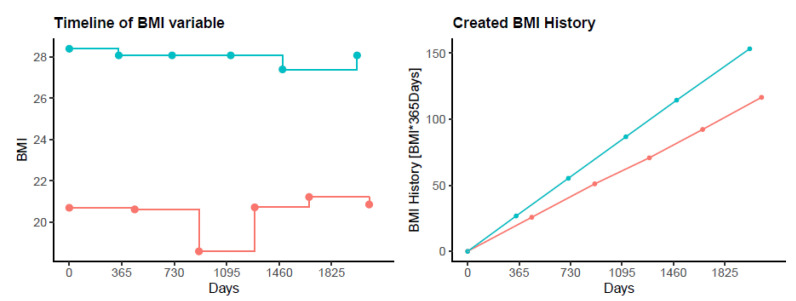
Example of the creation of a history variable. The longitudinally measured BMI variable presented on the left for two patients (red and blue) as an example for the construction of the history variable, which is presented on the right. The right plot represents the area under the curve from the left plot, summed up until the respective visits. The individual courses of BMI are therefore represented as the slope of the right curves.

All results are shown as mean values (standard deviation (SD)) or median (25%/75% quartiles) unless otherwise stated. An unconditional Kaplan–Meier curve was computed to plot overall mortality rate. Survival time was calculated as time between baseline visit and death, time to loss of follow-up, or time to last contact before work up for analysis. For each time-dependent variable, a corresponding history variable (sum of the products between the measurement of one visit multiplied by the time to next visit, see Figure 1) was constructed to investigate if there is an influence of the history of a variable on the current variable value. To test the assumption of the robustness of predictive values over time (whether effect estimates remain constant over time), multivariable left-truncated cox regression models with variables corresponding to the measurements at the respective visit were built for each study visit. Left-truncated Cox regression models including the history of the variable were performed to investigate if the histories of the variables have an influence on the estimated coefficients. Additionally, merged truncated Cox models and joint models out of all study visits as two methods for longitudinal modelling were built to assess the longitudinal predictive value of the variables on mortality. Joint modelling enables accounting for dropouts in longitudinal studies and covariates measured with error in time-to-event models can be included. Therefore, parameter estimate bias can be reduced. Each model consists of all predictors and was stratified by sex. Age at baseline was implemented as a time-independent continuous variable but presented as “per 10 year” unit for better interpretability. Finally, results from all models were compared with each other to check for outcome robustness. To avoid multiple testing bias, models were interpreted visually by their confidence intervals in a first step and possible predictors for mortality based on visual results and clinical knowledge were included in the final multivariable models. Statistical analysis was performed with R (R Core Team, 2020). A two-sided *p*-value of <0.05 was considered to be statistically significant.

## 3. Results

### 3.1. Study Participants

A total of 297 patients were included in the analysis and survival time was observed; 17 patients were lost to follow-up for survival during the study. 

For each study visit, the respective median time from baseline was calculated and only visits lying within a timeframe of a median ±3 months were included. Out of these 297 patient visits with baseline values, 173 remaining patient visits were analysed at visit 1, 109 at visit 2, 36 at visit 3, 10 at visit 4, and 5 at visit 5 (Figure S2). Patients who underwent lung transplantation during the study were excluded from the analysis. 

At baseline, mean (SD) age was 62.5 (7.6) years, 66% of the patients were males, and patients showed a mean (SD) BMI of 26.6 (6.1) kg/m2. A total of 71% of the patients were ex-smokers, whereas 25% were current smokers. Mean (SD) FEV_1_ % predicted was 48.8 (21.4)%. Detailed patient characteristics are shown in Table 1. 

### 3.2. Overall-Mortality

**Figure 2:**
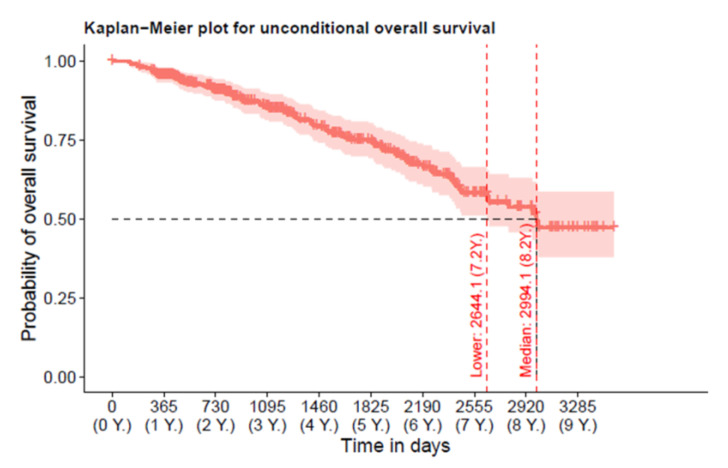
Kaplan–Meier plot for unconditional overall survival plus 95% confidence interval. Median (95% CI) survival time was 2994 (2644/NA) days (8.2 years). The upper bound lays outside of the observation period and is thus not defined.

The Kaplan–Meier plot shows unconditional overall survival (Figure 2). In total, 105 events were recorded with 48 (16.2%) deaths between baseline and visit 1, 22 (12.7%) deaths between visit 1 and 2, 13 (11.9 %) deaths between visit 2 and 3, 6 (16.7 %) between visit 3 and 4, and 1 (10.0%) death between visit 4 and 5. Median (95% CI) survival time was 2994 (2644/NA) days (8.2 years). A total of 35.4% of the patients died during the study period. 

### 3.3. Robustness of Predictors for Mortality over Time

Figure S1 shows that there is no evidence for a difference between the raw variable and the variable history on the predictive value for all tested variables over each visit (indicated by overlapping confidence intervals between raw and history variable). 

Furthermore, there was no evidence for changing effect estimates (coefficients) across the study visits due to the overlapping confidence intervals (Figure S1a–d), indicating changing variables over time with constant estimates and thus supporting the use of longitudinal models as appropriate.

Out of the results of Figure S1 and clinical knowledge on the relevant predictors of COPD mortality, age at baseline, 6MWD, FEV1 % pred., CAT, coronary artery disease, and pulmonary hypertension were included into the final multiple longitudinal models (a merged cox model with and without history and the joint model (Table 2)). 

**Figure 3:**
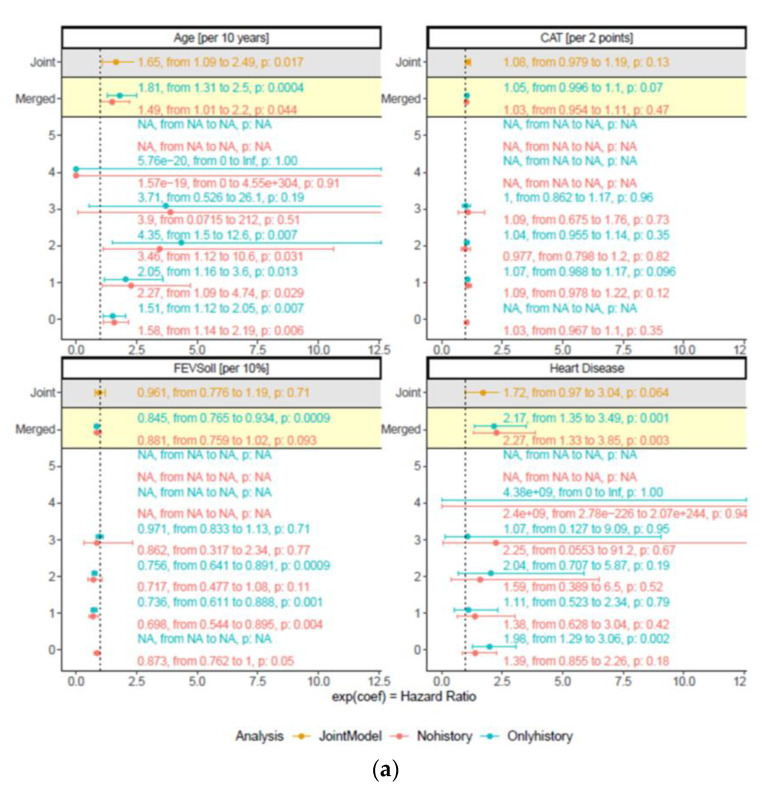

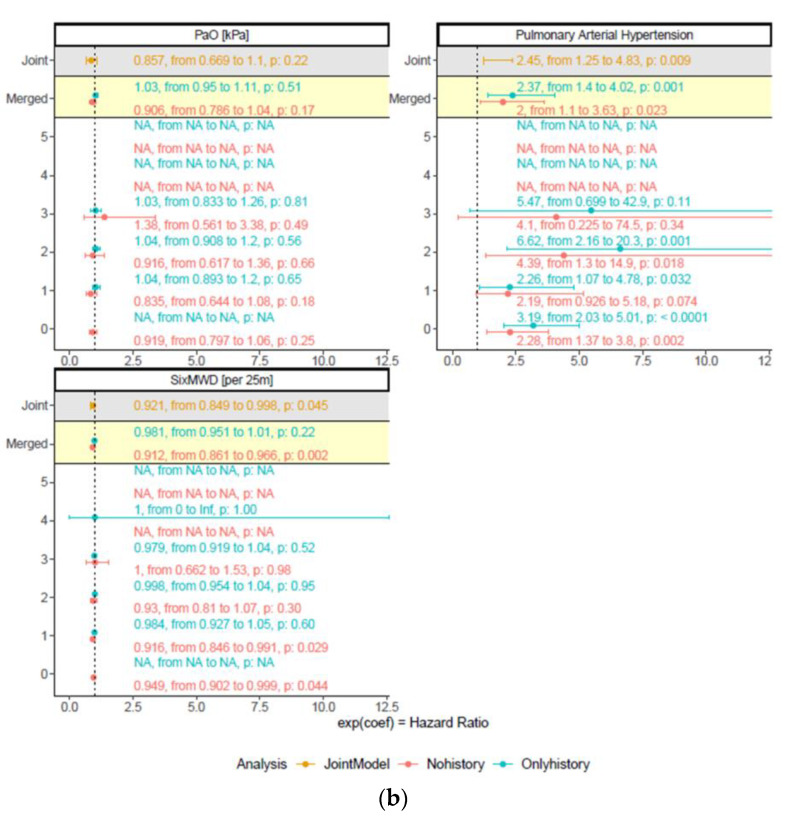
(**a**) Presentation of the variables age, CAT, FEV_1_ % pred., and heart disease of the final multivariable cox models. Results for each variable out of the three models are presented within one box (white, yellow, and grey areas). Analysis of prediction value for each visit (white area, visit 0–5), merged cox model (yellow area), and joint model (grey area) were used to compare predictor robustness over time. Non-history (red) and history-only (blue) values are presented for each visit (0–5) and the merged cox model. Results are presented as hazard ratio (95% confidence interval) and *p*-value. Age at baseline (presented as “per 10 years” unit) and pulmonary hypertension are independent, robust predictors of mortality. (**b**) Presentation of the variables PaO_2_, pulmonary hypertension, and 6 min walking distance of the final multivariable cox models. Results for each variable out of the three models are presented within one box (white, yellow, and grey areas). Analysis of the prediction value for each visit (white area, visit 0–5), merged cox model (yellow area), and joint model (grey area) were used to compare predictor robustness over time. Non-history (red) and history-only (blue) values are presented for each visit (0–5) and the merged cox model. Results are presented as hazard ratio (95% confidence interval) and *p*-value. Age at baseline (presented as “per 10 years” unit) and pulmonary hypertension are independent, robust predictors of mortality.

All three models showed for age at baseline (per 10 years) and pulmonary hypertension consistent results under the significance threshold of 0.05 and in the direction of the effect estimates. Furthermore, all three models showed comparable results to the results of the baseline visit, generating evidence for a robustness of effect estimates over time (Figure 3a,b).

## 4. Discussion

Although longitudinal studies reflect changes in disease characteristics over time, we found no evidence that predictors of mortality in COPD patients are time dependent. This implies that cross-sectional measured predictors show robust effect estimates over time and provide reliable measures in patients with COPD. 

For decades, mortality in patients with COPD and its predictors have been a widely investigated field of research. In the beginning, single physiological measures were postulated to cause the elevated risk of mortality in COPD patients such as COPD exacerbations [17], dyspnoea [18], or airway obstruction [17,18]. However, subsequent investigations showed that there rather seems to be an interaction of multiple factors than a single predictive factor. Considering this, Celli et al. [6] constructed a widely used predictive index, the BODE index. The index combines the multidimensional features body mass index (BMI), airway obstruction (FEV1 % pred.), dyspnoea (mMRC), and exercise capacity (6MWD) into an index score to identify impairment. The BODE index has been shown to be a good predictor for risk of hospitalizations [19] and mortality [20] in COPD and it seems to be a better predictor for mortality than airway obstruction as a single measure [21]. Furthermore, another multidimensional but simpler index, the ADO (age, dyspnoea, airflow obstruction) index, showed good prediction of 2-year mortality in COPD patients from primary care settings [22].

Henoch et al. [23] assessed the most important predictors for mortality in almost 40,000 COPD patients in a multivariable model and identified increasing age, lower FEV1 % pred., lower O_2_ saturation, worse dyspnoea, fewer days with exercise activity per week, and heart disease as independent predictors for increased all-cause mortality. According to the results of our study, increasing age and pulmonary hypertension might be the strongest independent predictors for all-cause mortality in COPD patients. However, the study by Henoch et al. [23] investigated possible predictors solely once in a cross-sectional setting. Since patient risk factors such as airway obstruction or symptoms may change in severity over time, it is of importance to evaluate if a single measure accurately reflects the predictive value of time-varying variables. We therefore analysed the postulated predictors from a longitudinal cohort with annual measurements to assess their robustness over time. Our results showed no evidence for a time-varying effect on the influence of possible predictors of mortality in COPD. The effect of a predictor on all-cause mortality seems to be robust over annual visits and therefore over time. Furthermore, there is no evidence that the history of a variable impacts the predictive value of the variable itself. Thus, our findings indicate that a single measure of possible predictors for mortality in COPD suffices and repeated measures do not enhance the predictive value on a population-based level. This is important finding as repeated measures are time- and cost-consuming, especially in the large sample studies investigating mortality. In contrast to Henoch et al. [23], we were not able to reproduce some of the postulated predictors for mortality such as FEV_1_% pred. or heart disease in our study. This may be because of the lower sample size in our study. However, the direction of possible predictors (favour/non-favour) is comparable and since the focus of the paper was to investigate the robustness of the predictors over time this does not affect the outcome.

The study has some limitations. We were not able to ascertain the causes of death and therefore we cannot conclude the influence of COPD on the mortality rates. However, the cohort represents a real-world COPD population with its typical comorbidities. Due to the decreasing sample size for the later visits, time-dependent predictors cannot be excluded for all visits. Since the predictors seem consistent over the first three visits, we assume consistency over the later visits. However, the results provide preliminary evidence for time consistency and it remains to be investigated if longitudinal assessments over more than 5 years yield a time-varying effect for known predictors of overall mortality, since COPD is a disease mostly progressing over decades with different disease trajectories and potential development of new comorbidities. Furthermore, the end of study was set as an arbitrary time point and not all patients were followed-up until death. 

## 5. Conclusions

To the best of our knowledge, this is the first study investigating the effect of time-varying predictors for mortality in COPD. There is no evidence for a time-varying effect in the possible predictors of all-cause mortality in COPD patients. Therefore, multiple or annual assessments of predictors for mortality seem not to change the predictive value of the measure. This finding is of relevance for the mostly large survival studies in COPD patients, since repeated measurement of possible predictors seems not to be beneficial. Despite that finding, longitudinal settings are crucial to investigate treatment effects and evolution of diseases.

## Figures and Tables

**Table 1:** Subject characteristics.

	N = 297
Age, y	62.5 (7.6)
Female/Male, N	101/196
BMI, kg/m^2^	26.6 (6.1)
*Comorbidities*	
Arterial hypertension, N (%)	140 (47)
Coronary artery disease, N (%)	55 (19)
Heart failure, N (%)	23 (8)
Arteriosclerosis, N (%)	83 (28)
Lung cancer, N (%)	4 (1)
Cor pulmonale, N (%)	12 (4)
Pulmonary hypertension, N (%)	36 (12)
Chronic renal failure, N (%)	17 (6)
Diabetes, N (%)	39 (13)
*Smoking history*	
Never smokers, N (%)	11 (4)
Current smokers, N (%)	74 (25)
Ex-smokers, N (%)	212 (71)
Pack years of smoking, N	45.6 (27.7)
*Respiratory variables*	
FVC, % pred.	81.2 (20.7)
FEV_1_, % pred.	48.8 (21.4)
RV/TLC, %	54.9 (11.8)
PaO_2_, kPa	9.1 (1.6)
CAT score	15 (11/21)
mMRC score	2.0 (1.0/2.0)
Number of exacerbations in the previous year, N	1.0 (1.4)
6MWD, m	415.7 (130.1)

Values are mean (SD) or median (25%/75% quartiles) unless otherwise stated. BMI: body mass index; FVC: forced vital capacity; pred.: predicted; FEV_1_: forced expiratory volume in 1 s; PaO_2_: partial oxygen pressure; CAT: COPD assessment test; mMRC: modified medical research council; 6MWD: 6 min walking distance.

**Table 2:** Multivariable joint model.

	Coef. (95% CI)	*p*-Value
Age at baseline, per 10 year	1.65 (1.09/2.49)	0.017
CAT	1.08 (0.98/1.19)	0.13
FEV_1_, % pred.	0.96 (0.78/1.19)	0.71
Coronary artery disease	1.72 (0.97/3.04)	0.064
PaO_2_, kPa	0.86 (0.67/1.10)	0.22
Pulmonary hypertension	2.45 (1.25/4.83)	0.009
6MWD, m	0.92 (0.85/0.998)	0.045

CAT: COPD assessment test; FEV_1_: forced expiratory volume in 1 s; PaO_2_: partial oxygen pressure; 6MWD: 6 min walking distance.

## Data Availability

The datasets used and analysed during the current study are available from the corresponding author on reasonable request.

## Supplementary Materials

The following supporting information can be downloaded at: https://www.mdpi.com/article/10.3390/jcm12041587/s1, Figure S1a–d: Results from the separately fitted multivariable cox models for each visit (white area) and merged cox models (yellow area) were shown. Results are presented as hazard ratio (95% confidence interval) and *p*-value, Figure S2: Patients journey on the left side. Filtered visits happening in predefined timeframes ± 3 months on the right side.
