# Supplementary material for: Are Predictors for Overall Mortality in COPD Patients Robust over Time?
Source: J Clin Med. 2023 Feb 16;12(4):1587. doi: 10.3390/jcm12041587 (PMC9961228; doi:10.3390/jcm12041587)
Supplement: Supplementary file 1 [file jcm-12-01587-s001.zip › jcm-2058840-supplementary.pdf]

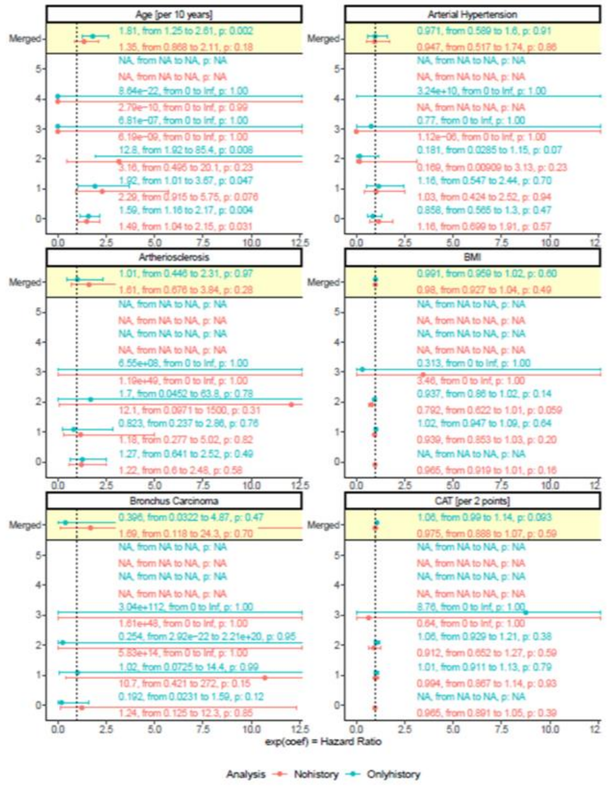
Age [per 10 years]
Arterial Hypertension
Artheriosclerosis
BMI
Bronchus Carcinoma
CAT [per 2 points]
exp(coef) = Hazard Ratio
Analysis Nohistory Onlyhistory

(**a**)

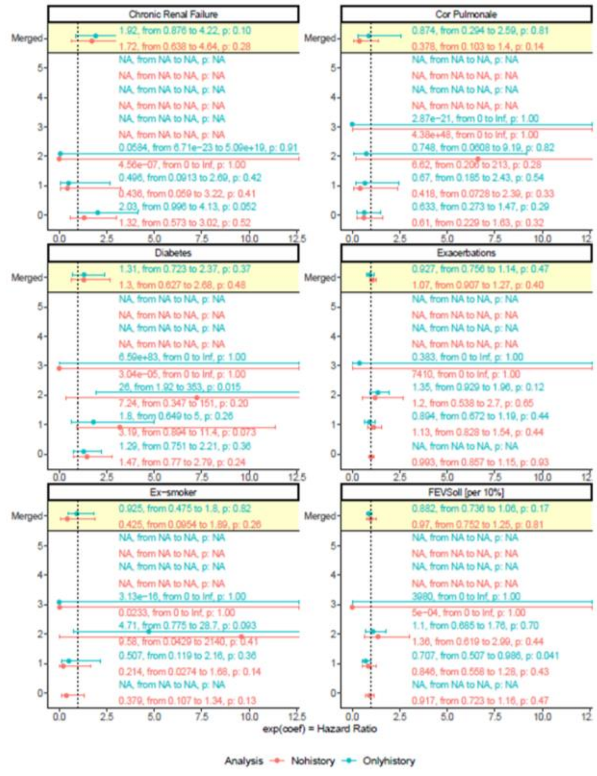
Chronic Renal Failure
Cor Pulmonale
Diabetes
Exacerbations
Ex-smoker
FEVSoll [per 10%]
exp(coef) = Hazard Ratio
Analysis Nohistory Onlyhistory

(**b**)

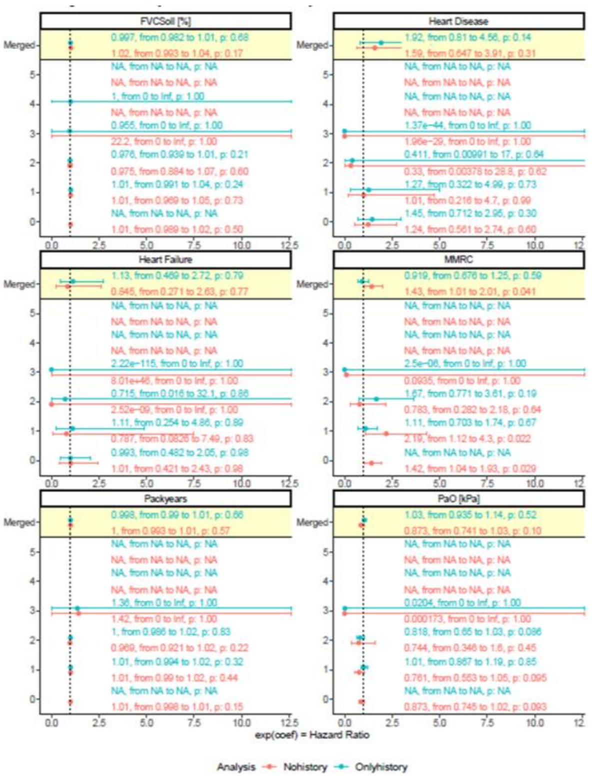

(**c**)

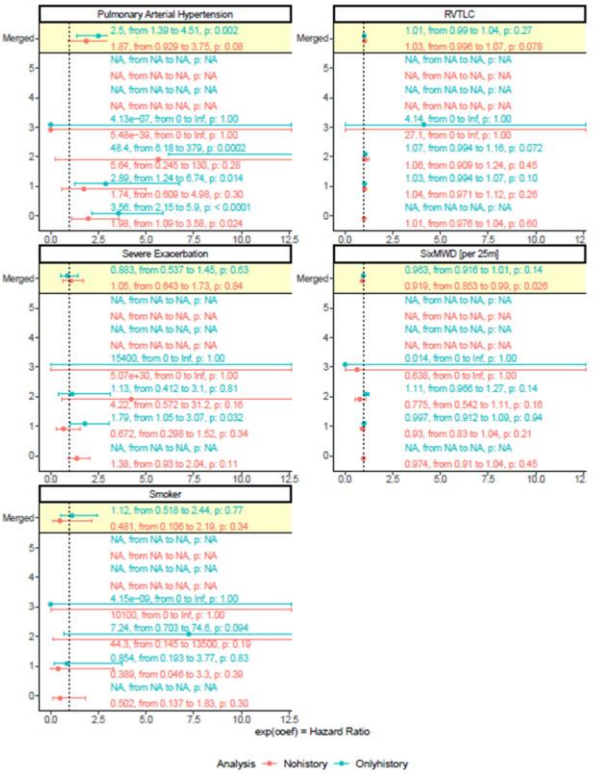

(**d**)

Figure S1. (**a–d**) Results from the separately fitted multivariable cox models for each visit (white area) and merged cox models (yellow area) were shown. Results are presented as hazard ratio (95% confidence interval) and p-value.

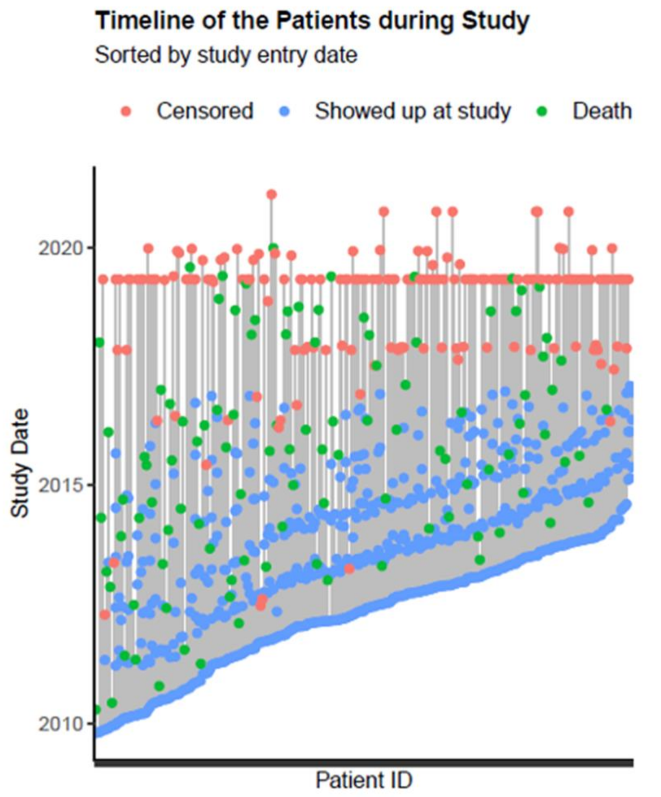

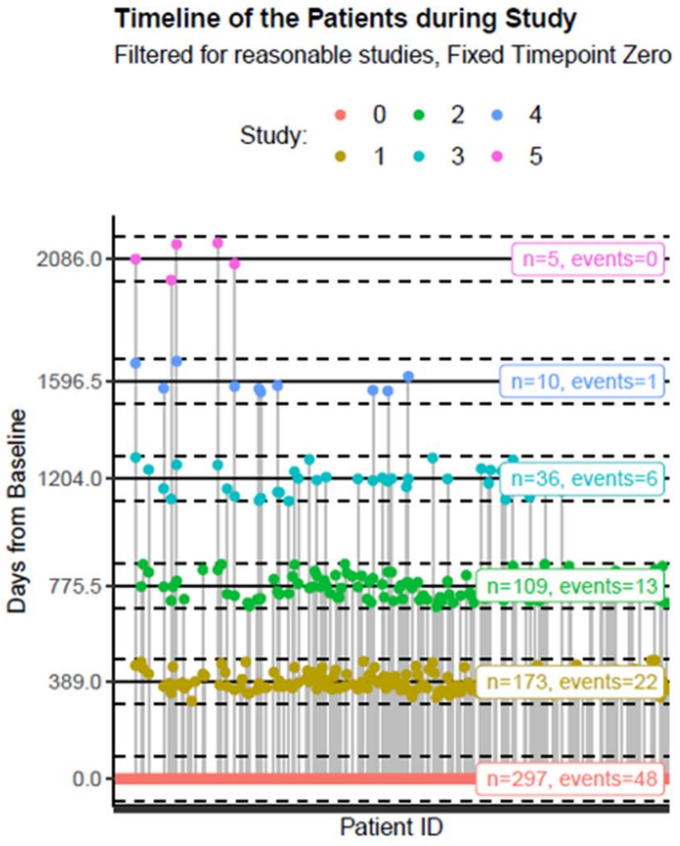

Figure S2. Patients journey on the left side. Filtered visits happening in predefined timeframes ± 3 months on the right side.
